# A COVID Spring 2020: a reflection

**DOI:** 10.1017/ipm.2020.34

**Published:** 2020-05-14

The Twenties did not roar,

They entered with a silent scream.

There is a new/old anthem for this time,

‘The Sound of Silence’.

for not 10,000 but 45,000 and so many more.

People seeing without perceiving,

People listening but not hearing,

People speaking but not thinking.

However, silent words captured by philosophers and psalms.

In Chinese tenements,

Italian piazzas

Spanish gardens

French boulevards,

English brick houses,

Irish care homes,

And now American landscapes.

A new Dust Bowl of memories,

tumbling like sweets from a jar

or floating as raintears from the sky.

It is a time that will not be trumped

by pernicious Fake news.

It is a time for us to cherish,

those who were lost,

and those who served.

It is a time to bear witness to this silence,

feeling it’s poignant music.

The sound of our lives our loves

This Spring of two thousand and twenty.

*Kieran Darragh O’Malley*

*March 30/31 2020*,

*Carnalea (in lockdown)*

*Honouring Paul Simon and Art Garfunkel*.

